# Role of Nivolumab in the Modulation of PD-1 and PD-L1 Expression in Papillary and Clear Cell Renal Carcinoma (RCC)

**DOI:** 10.3390/biomedicines10123244

**Published:** 2022-12-13

**Authors:** Joanna Bialek, Stefan Yankulov, Felix Kawan, Paolo Fornara, Gerit Theil

**Affiliations:** Medical Faculty, Clinic of Urology, Martin Luther University Halle-Wittenberg, 06120 Halle (Saale), Germany

**Keywords:** renal carcinoma, RCC, PD-1, PD-L1, nivolumab

## Abstract

The expression and cellular mechanisms of programmed cell death-1 protein (PD-1) and its ligands (PD-L1 and PD-L2) in renal cancer cells are not well known. Here, we aimed to investigate the response of renal carcinoma subtypes to the immune checkpoint inhibitor nivolumab and its impact on related signaling pathways. All cell lines analyzed (clear cell (cc)RCC (Caki-1, RCC31) and papillary (p)RCC (ACHN, RCC30)) expressed PD-1 and both ccRCC cell lines, and RCC30 expressed PD-L1. Nivolumab treatment at increasing doses led to increased PD-1 levels in analyzed cells and resulted in aggressive behavior of pRCC but diminished this behavior in ccRCC. The analysis of PD-1/PD-L1-associated signaling pathways demonstrated increased AKT activity in Caki-1 and RCC30 cells but decreased activity in ACHN and RCC31 cells, while ribosomal protein S6 remained largely unchanged. Androgen receptors are related to RCC and were predominantly increased in RCC30 cells, which were the only cells that formed nivolumab-dependent spheroids. Finally, all cell lines exhibited a complex response to nivolumab treatment. Since the pRCC cells responded with increased tumorigenicity and PD-1/PD-L1 levels while ccRCC tumorigenicity was diminished, further studies are needed to improve nivolumab-based therapy for renal carcinoma subtypes, especially the identification of response-involved molecular pathways.

## 1. Introduction

Kidney cancer is one of the most common cancers, as more than 403,000 new cases are diagnosed each year, which accounts for 2.2% of all newly diagnosed cancers worldwide [1]. The two most common variants, clear cell renal cell carcinoma (ccRCC) and papillary renal cell carcinoma (pRCC), originate from proximal tubule epithelial cells and display morphological differences [2,3]. The clinical prognosis of patients with pRCC is better than that of patients with ccRCC, who may develop metastasis to the lung, liver, bones or lymph nodes [2,3].

Tumor expansion is complex. The development of immune checkpoint inhibitors (ICIs) has transformed therapeutic options in oncology by inhibiting the survival of tumor cells through the escape from immunological destruction. Nivolumab, an agent that blocks the interaction between PD-1 and its ligands (PD-L1 and PD-L2), is approved for the treatment of many cancers, including renal carcinoma (RCC). As with other therapies, many patients experience a good quality of life during treatment; however, some patients can experience immune-related adverse events, which can induce atypical responses, such as hyperprogressive disease or pseudoprogressive disease, and increase the mortality risk [4,5,6]. To improve quality of life, the efficacy of combined therapy (example TKI/PD-1/PD-L1) was tested [7].

For a long time, it was believed that the PD-1 expression was limited to mature cytotoxic T lymphocytes, while its ligands were primarily expressed by tumors. Recently, Wang et al. [8] described an analysis of the intrinsic expression of PD-1 in different tumors, including renal carcinoma. Little information has been reported on the signaling pathways of PD-1/PD-L1 molecules. As reviewed by Han et al. [9], the silencing of cell-intrinsic PD-1 results in higher proliferation of the non-small cell lung cancer cell lines NCI-H1299 and Calu-1 as well as increased phosphorylation of protein kinase B (AKT) (p-AKT) and extracellular-signal regulated kinase (ERK1/2) (p-ERK). This suggests the involvement of pathways similar to those of T-cells [8] and indicates that intrinsic PD-1 is a potential tumor suppressor [9]. In melanoma cells, PD-L1 signaling is correlated with AKT and S6 protein phosphorylation, which suggests the activation of the mTOR pathway.

The incidence of RCC is higher in men than in women, which implies the participation of steroid hormones in tumor development. It has been suggested that supportive treatment based on steroid receptor signaling pathways should be considered for renal carcinoma [10]. Expression of androgen receptor (AR) was analyzed in many tumors [11,12,13,14,15,16] supplying information about tumor growth, survival time [12] and advancement for antiandrogen therapy [17]. The relationship between AR and RCCs was analyzed in many studies. Among others, AR has been considered as a marker of indolent RCC and is associated with tumor-suppressive activity [10]. However, its role in RCC is still controversial. In their review, Czarnecka et al. [10] discuss the utility of AR in the prognosis, diagnosis and treatment of RCC. The authors describe AR as a favorable marker in RCC that is correlated with a low stage or grade as well as with primary tumor tissues rather than metastases. However, many reports present opposed results [18,19,20]. In our previous studies, we found that AR was differentially expressed in different types of RCC [21].

In many gender disparity tumors, such as HCC or thyroid, AR bind the PD-L1 promotor and so downregulates PD-L1 expression [22,23]. Guan et al. [24] noted that blocking AR and PD-L1 correlated with a reduction in prostate tumor growth, which provides a connection between androgens and therapy resistance. Additionally, cathepsin B (Cath B) is correlated with therapy resistance [25], and overexpression of this protease is associated with invasive phenotypes of many cancers [26]; moreover, this protein has been demonstrated to have a tumor-protective role [27]. This is why its expression was analyzed as a marker of the effectiveness of several therapies [26].

In our study, we investigated the expression of PD-1 and PD-L1 in two ccRCC and two pRCC cell lines and the functional response of these cells to the immune checkpoint inhibitor nivolumab. Furthermore, we tested the expression of proteins such as AR as well as the typical ones of signaling pathways that were previously identified as related to PD-1/PD-L1 cascades.

## 2. Materials and Methods

### 2.1. Cell Culture

The human papillary renal carcinoma cell line ACHN and the ccRCC cell line Caki-1 obtained from American Type Culture Collection (ATCC, Manassas, VA, USA) were cultured in RPMI medium (Life Technologies Europe B.V., Bleiswijk, The Netherlands); the cell lines established from RCC tissues (the papillary RCC30 cell line and the clear cell RCC31 cell line) were obtained from the Institute of Medical Immunology MLU Halle and were cultured in glucose-high DMEM (Life Technologies). All media were enriched with 10% fetal calf serum (FCS) (Capricorn Scientific GmbH, Ebsdorfergrund, Germany), and the primary RCCs were enriched with MEM Non-Essential Amino Acid Solution (Gibco/Life Technologies Europe B.V.). All cells were cultured at an early passage. The cells were passaged every 4–5 days, and the culture medium was changed every 2–3 days. Prior to experiments, the cells were seeded in 6- or 96-well plates (Sarstedt, Nümbrecht, Germany) and allowed to adhere for 24 h. For each experiment, the cells were incubated with increasing concentrations of nivolumab (0, 12, 24, 36, 48 or 72 µg/mL), which should correspond to the monotherapy used as second-line therapy for RCC. Our experiments were performed in a monoculture (immune-independent conditions). The initial nivolumab concentration corresponds with that used in patients. Higher levels of the ICI were used to test the signals in cancer cells.

### 2.2. RNA Isolation/RT- and q-PCR

After one day of growth in 6-well plates (Sarstedt), the cells (1 × 10^6^) were incubated with nivolumab (OPDIVO, Bristol-Myers Squibb, Berlin, Germany) (0, 12, 24, 36, 48, 72 µg/mL) for 24 or 48 h. Subsequently, all cells were scraped off, and RNA was isolated using an RNA isolation kit (RNeasy Plus Mini Kit, Qiagen, Hilden, Germany). cDNA was synthesized using a SuperScript IV VILO Master Mix (Thermo Fisher, Dreieich, Germany) kit at 42 °C for 15 min according to the manufacturer’s instructions. Quantitative PCR (qPCR) was performed using 5× Hot FirePol Eva Green qPCR Mix Plus (Solis Biodyne, Tartu, Estonia) in a QuantStudio5 Thermocycler (Thermo Fisher Scientific, Waltham, MA, USA). The expression of the target genes was analyzed using specific primers, and β-actin served as an endogenous control (Table 1). The results were calculated using the 2-ΔΔCT method.

### 2.3. Western Blot Analysis

A total of 10 µg of protein isolated with RIPA buffer (Cell Signaling, Leiden, The Netherlands) was separated in 4–12% polyacrylamide gels (Thermo Fisher), followed by a 1 h transfer at 350 mA 4 °C. The membranes were incubated in blocking solution (5% BSA/TBS-T 0.01%) for 1 h and with the primary monoclonal antibodies (PD-1, PD-L1, AKT, pAKT, S6 and pS6 rabbit antibodies all from Cell Signaling; β-actin (mouse) from Sigma, Burlington, MA, USA) overnight at 4 °C. The membranes were then incubated with the corresponding secondary antibodies (peroxidase-labeled anti-rabbit or anti-mouse antibodies) for 1 h at room temperature. The bands were visualized with Lumi-Light Western Blotting Substrate (Roche, Manheim, Germany) in a ChemiDoc™ Touch Imaging System (Bio-Rad, Feldkirchen, Germany).

### 2.4. Cell Viability Assay

One day after the cells (1 × 10^4^/well) were seeded in a 96-well plate (Sarstedt), the growth medium was exchanged for medium containing increasing concentrations of nivolumab (0, 12, 24, 36, 48, 72 µg/mL) after which the cells were cultured for 24 or 48 h. A WST assay (Merck/Sigma) was performed according to the manufacturer’s instructions. After 4 h of incubation at 37 °C, the absorbance was measured at 450 nm using a Cytation5 instrument (Agilent Technologies, Waldbronn, Germany).

### 2.5. Scratch Assay

We seeded the cells in a 6-well plate and allowed them to grow until confluent. The monolayer was scratched with a sterile 100 µL pipette tip in the center of the well, and the growth medium was discarded. After gentle washing in PBS (Sigma), the cells were incubated with a control medium or a medium containing different concentrations of nivolumab (12, 24, 36, 48, 72 µg/mL). The 24 h assay was performed with a Cytation5 instrument (Agilent Technologies), and images were obtained every 2 min. After one day, the final images were analyzed.

### 2.6. Spheroid Formation

The 3D culture was initiated using 1 × 10^4^ cells/well in a nonadherent round-bottom 96-well plate (Sarstedt). The control medium and medium enriched with nivolumab were changed every 4–5 days. Spheroid formation was observed for 6 weeks. Images and spheroid measurements were performed using a Cytation5 reader (Agilent Technologies). The aggregates were considered spheroids if they displayed visible 3D structures.

### 2.7. Cath B Activity

Cath B activity was measured using a fluorometric Cathepsin B Activity Assay Kit (Abcam, Berlin, Germany). All cells (1 × 10^5^/well) were seeded in 6-well plates (Sarstedt) and treated with increasing concentrations of nivolumab the following day. After 24 h of incubation, the cells were harvested, washed in PBS (Sigma) and resuspended in chilled Cell Lysis Buffer (4 °C), which was included in the kit, for 30 min, followed by centrifugation at 4 °C for 5 min. The activity was measured in the supernatant according to the manufacturer’s instructions. The output was measured using a Cytation5 reader (Agilent Technologies) at Ex/Em = 400/505 nm.

## 3. Results

### 3.1. Intrinsic Expression of PD-1 and PD-L1

**Figure 1:**
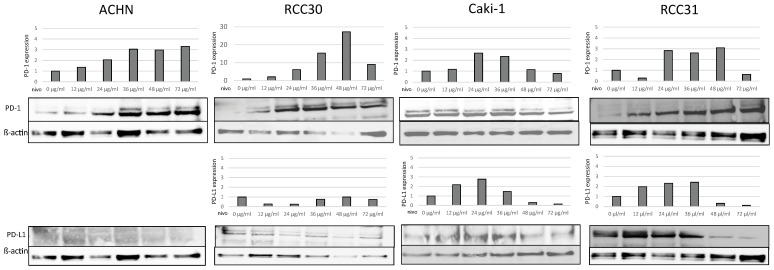
Expression of PD-1 and PD-L1 in renal carcinoma cell lines. Caki-1, RCC30, RCC31, but not ACHN cells, expressed PD-L1 when PD-1 was expressed in these cells.

We first analyzed the expression of PD-1 and PD-L1 in all four RCC cell lines. The Western blots performed on cell lysates revealed bands with a size of approximately 55 kDa, which corresponds to PD-1, and >55 kDa, corresponding to PD-L1 (Figure 1). We observed an increase in the PD-1 expression in papillary cell lines after nivolumab treatment. The expression of PD-L1 was either weak (RCC30) or absent (ACHN). In ccRCC cells, the PD-L1 expression was increased after treatment with low concentrations of nivolumab (12–36 µg/mL) and was decreased after treatment with higher concentrations (48–72 µg/mL). PD-1 expression was increased in both ccRCC—Caki-1 (24–36 µg/mL) and RCC31 (24–48 µg/mL) cells and was decreased with the highest nivolumab concentrations, while in pRCCs was increased.

### 3.2. AKT and S6 Activation

**Figure 2:**
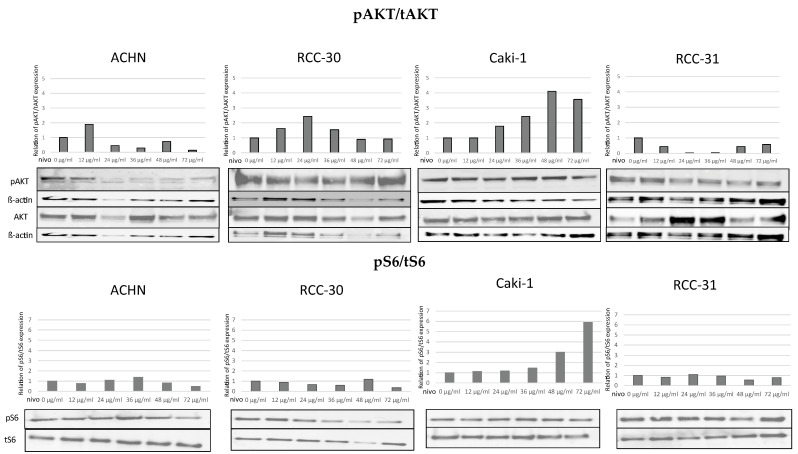
Status of AKT and S6 activation. The relationship between p/t expression of tAKT and pAKT as well as S6/pS6 in RCC cell lines was defined as the protein activation status.

We analyzed the expression of phosphorylated (p) and total (t) AKT and S6 (Figure 2). We considered the relationship between p and t (p/t) as an indicator of protein activation. Papillary cell lines (ACHN, RCC30) reacted positively at low concentrations of nivolumab, while increasing amounts decreased AKT activity. The results obtained for ccRCC cells are inconsistent. AKT activity was increased under the influence of nivolumab in Caki-1 cells, while this activity was decreased in RCC31 cells. In addition, besides the increase induced in Caki-1 cells, no significant differences were observed in S6 protein activation.

### 3.3. Contribution of Nivolumab to Metabolic Activity

**Figure 3:**
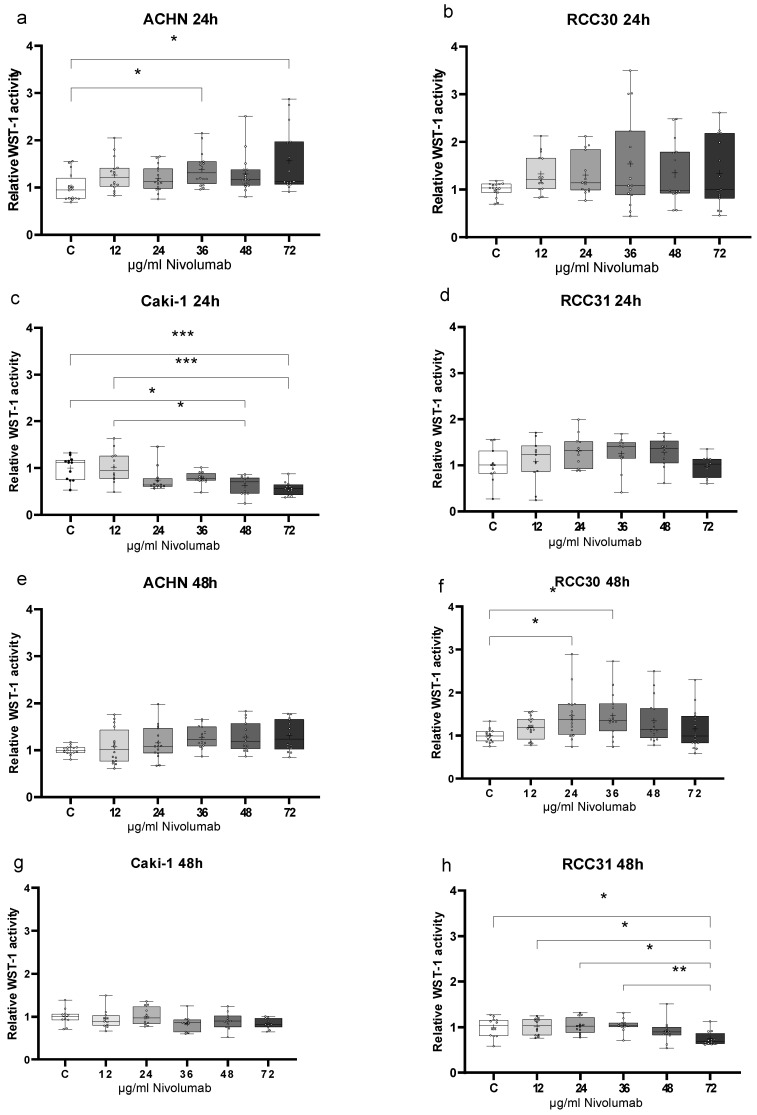
Metabolic activity of renal carcinoma cell lines. WST-1 activity was measured after 24 h (**a**–**d**) and 48 h (**e**–**h**) in papillary (**a**,**b**,**e**,**f**) and clear cell (**c**,**d**,**g**,**h**) renal carcinoma cells. * ≤ 0.05, ** ≤ 0.01, *** ≤ 0.001.

Using a WST assay, we analyzed the influence of nivolumab on the viability of renal carcinoma cells. The results revealed a significant response of ACHN and Caki-1 cells after 24 h (Figure 3a–d), while the tissue-derived cell lines reacted after 48 h (Figure 3e–h). Both papillary cell lines (ACHN, RCC30) responded with an increase in metabolic activity in the presence of nivolumab (Figure 3a,f). The increasing trend in ACHN cells was noted at all concentrations; however, significant differences were observed at concentrations of 36 µg/mL and 72 µg/mL (both *p* > 0.05) (Figure 3a). RCC30 cells responded with a significant increase in metabolic activity at concentrations of 24 µg/mL and 36 µg/mL (*p* > 0.05), while higher concentrations decreased the metabolic activity to the baseline level (Figure 3f). Notably, nivolumab diminished the metabolic activity of ccRCC cell lines only at higher concentrations (Figure 3c,h). Caki-1 cells exhibited significant differences at 48 µg/mL (*p* > 0.05) and 72 µg/mL (*p* > 0.001) (Figure 3c), while RCC31 cells exhibited significant differences at only 72 µg/mL (*p* > 0.05 and 0.01) (Figure 3h).

### 3.4. Motility of Renal Carcinoma Cell Lines

**Figure 4:**
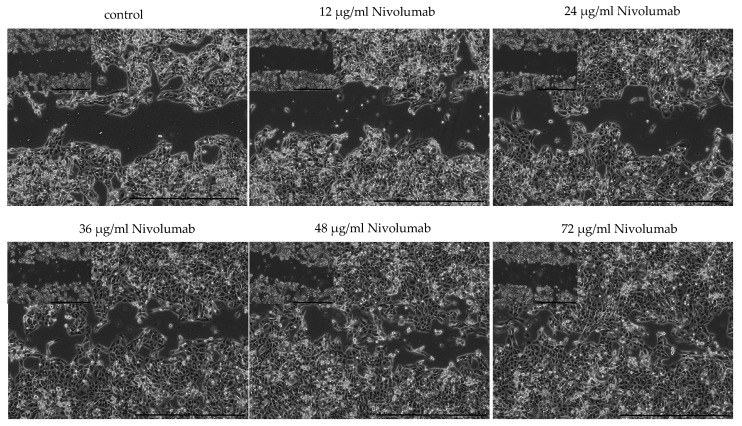
Motility of ACHN cells treated with nivolumab for 24 h; scale bars 1000 µm.

Scratch assays were performed to examine the influence of nivolumab on cell motility. As demonstrated in Figure 4, ACHN cells reacted to the treatment by almost complete closure of the scratched area (Figure 4). The primary cell line RCC30 partially responded with increased cell motility after treatment with low concentrations of nivolumab (12–36 µg/mL). Differences in the responses of Caki-1 and RCC31 cells were not detected (not shown).

### 3.5. Spheroid Formation

**Figure 5:**
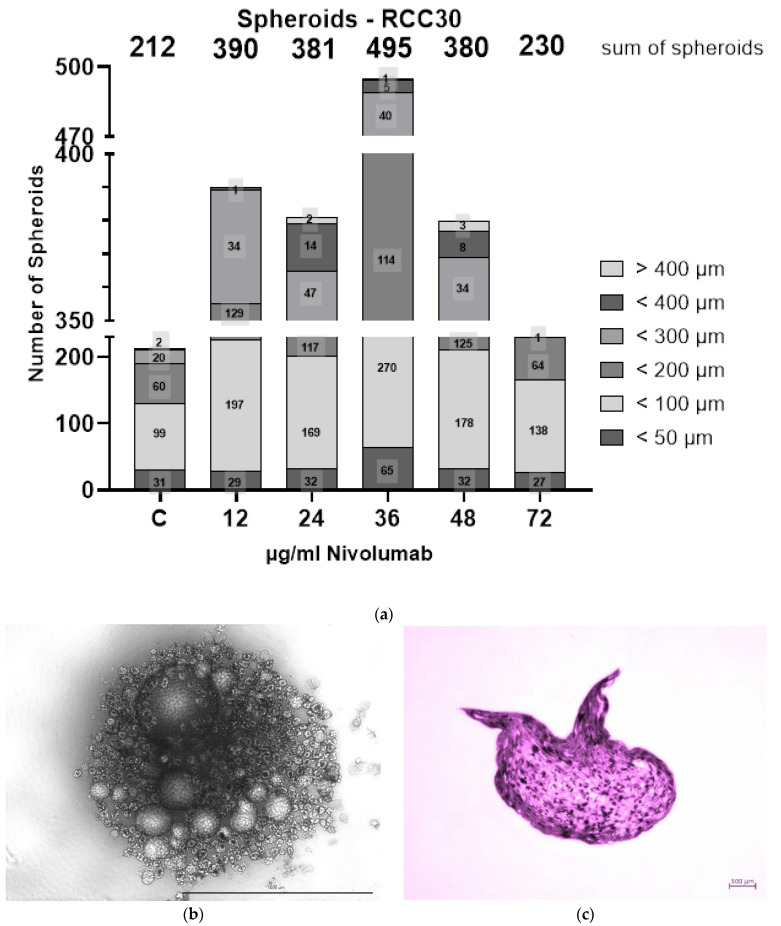
3D culture under nivolumab treatment. Number of spheroids formed by the RCC30 cell line (**a**). Spheroids formed by the RCC30 cell line under bright field microscopy; the scale bar: 1000 µm (**b**) and after HE staining; the scale bar: 100 µm (**c**) at a concentration of 36 µg/mL nivolumab.

The only cell line that formed spheroids upon nivolumab treatment was RCC30 (Figure 5a–c). The spheroids were detected at every concentration of nivolumab as well as in the control group. The numbers of spheroids in the control group and in the group treated with 72 µg/mL nivolumab were similar at 212 and 230, respectively, while the numbers were higher at concentrations of 12 µg/mL (390 spheroids), 24 µg/mL (381 spheroids) and 48 µg/mL (380 spheroids) (Figure 5a). The highest number (495) of spheroids was observed at 36 µg/mL nivolumab. Most spheroids in each group were relatively small, reaching up to 100 µm in diameter. The highest number of them was observed at a concentration of 36 µg/mL nivolumab (335 spheroids) compared with the control (130 spheroids). It is worth noting that spheroids greater than 400 µm were formed only at concentrations that ranged from 24–48 µg/mL (Figure 5a). During the six weeks, ACHN, Caki-1 and RCC31 cells formed aggregates of similar size but not regular spheroids, which were independent of nivolumab concentration.

### 3.6. Cath B Expression and Activity

**Figure 6:**
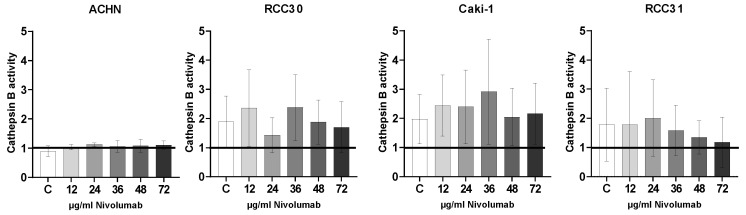
Cath B in RCC cells. Cath B activity after incubation with increasing concentrations of nivolumab. The activity in each cell line after using a specific inhibitor was defined as “1”.

Nivolumab did not influence the expression of Cath B in any of the cell lines (not shown). We analyzed the activity of Cath B by normalizing the results after employing the specific inhibitor (included in the kit) and defining its results as “1”. Cath B activity was detected in three (RCC30, Caki-1 and RCC31) of four cell lines. The ACHN cell line showed levels equal to when the inhibitor was used. Exposure to nivolumab for 24 h had no significant influence on Cath B activity (Figure 6).

### 3.7. AR Expression

**Figure 7:**
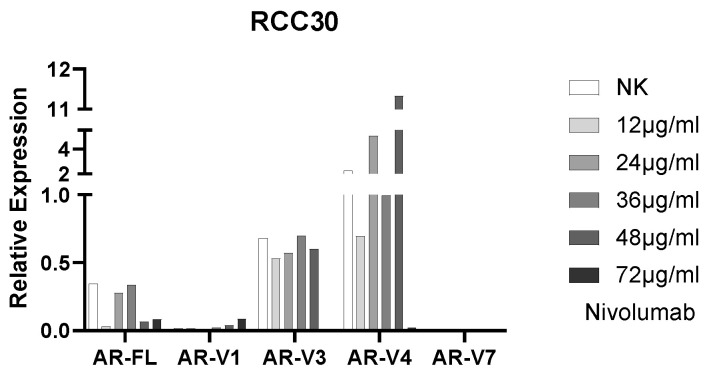
Expression of AR-FL and AR-SVs in RCC30 cells.

In our study, we noticed differences with regard to tumor type, as papillary tumor cells showed a higher expression of AR-FL (full length) and AR-SVs (splice variants) than ccRCC cells (Figure 7). Considering the results of the most well studied variants AR-FL and AR-V7 obtained in naïve cells, AR-FL was detected only in primary cells, while AR-V7 SV was present in ccRCC cells only. The three additional variants displayed similar patterns among all tested cell lines, with minimal detection of AR-V1, higher AR-V3 and the highest detection of AR-V4. Generally, except for the primary papillary RCC30 cell line, the expression of all ARs was weak in all tested cell lines (not shown). Incubation with increasing concentrations of nivolumab did not influence AR expression.

## 4. Discussion

This study clearly demonstrated the effects and pathways that might be involved in the response to nivolumab treatment in clear cell and papillary RCC cell lines. In an immune-independent manner, we observed that, in contrast to clear cell RCC cells, papillary RCC cells responded to nivolumab with increased aggressive behavior manifested as a dose-dependent response, higher motility and higher metabolic rates.

Treatment of RCC patients is a challenge because approximately one-third of RCC patients already present with metastasis at the initial diagnosis. After surgical resection, another one-third experience recurrence with distant metastases [3]. The management of ccRCC patients with immunotherapies shows positive effects, but knowledge of advances in nonclear cell RCC is limited.

The indication for nivolumab immune therapy is PD-L1 expression. However, the phase 3 CheckMate 025 trial demonstrated that even if the expression of PD-L1 (≥1%) in RCC is associated with poorer survival, the benefit of therapy is independent of PD-L1 status. The PD-L1 as a marker for the treatment outcomes seems to be dependent on tumor type or histologic class [30].

PD-1 was described for the first time in murine hematopoietic progenitor and murine T- hybridoma cell lines in 1992 [31]. As reviewed by Han et al. [9], this immune response inhibitor is present in activated T-cells, natural killer cells and B lymphocytes as well as macrophages, dendritic cells and monocytes. PD-1 binds PD-L1 on cancer cells and initiates the immune escape of tumor cells. This process can be blocked with immune checkpoint inhibitors, such as nivolumab, which bind PD-1 and prevent the interaction with PD-L1.

Recently, intrinsic tumor expression of PD-1 was described in various cancer cells [32,33,34], including renal cancer [8]. Wang et al. [8] demonstrated PD-1 transcripts in four renal cancer cell lines (ACHN, Caki-1, 769-P, 786-O). In our study, we observed PD-1 protein in ACHN and Caki-1 cells as well as in the two in-house established cell lines RCC30 (pRCC) and RCC31 (ccRCC).

In papillary RCC, PD-1 expression is constant when PD-L1 expression is weak (RCC30) or absent (ACHN). In contrast, the clear cell carcinoma cell lines Caki-1 and RCC31 strongly express both proteins. Our study shows that exposure of tumor cells to increasing concentrations of nivolumab seems to downregulate the PD-1 expression and partially downregulate PD-L1 in ccRCC, but seems to increase PD-1 expression in pRCC. This partly coincides with our analysis of the metabolic activity in cells treated with nivolumab. At high concentrations, we observed a reduction in WST-1 generation in ccRCC cells but an increase in pRCC cells. The results obtained in our study suggest the activation of intrinsic PD-1 without the necessary involvement of PD-L1 in papillary RCCs. This lead probably to the initiation of still not known signaling cascades, which induce aggressive behavior of the cells. Lately, the results of open-label phase IIIb/IV CheckMate 374 study established monotherapy with nivolumab as the treatment option for advanced nccRCC. From the pRCC patients of the cohort and only one demonstrated partial response (PR), 9—stable disease but 11—progressive disease [35]. In other studies, in combination with ipilimumab treatment (anti-CTLA-4 antibody) also only 1/18 pRCC patients responded complete (CR) for the treatment, when 4/18 were PR [36]. Du et al. [33] observed that the NSCLC cell line M109 xenotransplanted into mice demonstrated increased viability associated with exposure to anti-PD-1 blockade. Kleffel et al. [32] and Wang et al. [8], however, noticed suppression of melanoma and lung tumor cell growth, respectively, after antibody blockade. Interestingly, the PD-1 protein differed significantly in the size and number of detected bands (~55 kDa [8] and ~32 kDa [32]) between the two studies.

The results of Denize et al. [37] raise the possibility that PD-L1 expression in ccRCC cancer cells might require intrinsic pro-oncogenic signals from tumor cells. When coexpressed in tumors, PD-L1 can activate intrinsic PD-1 in an immune-independent manner and thus modulate signaling cascades in tumor cells [38]. Considering the differential intrinsic expression and potentially differential PD-1/PD-L1 interaction intensity within the two (clear and papillary) types of renal carcinoma, one could speculate the activation of different signaling pathways (PI3K/AKT, MAPK, mTOR).

The PI3K/AKT/mTOR signaling pathway is implicated in the development of many human malignancies, including RCC [37]. The PD-1/PD-L1 cascade is thought to be involved in the initiation of mTOR signaling. Intrinsic PD-1, activated by PD-L1, activates mTOR [38], while the inhibition of mTOR increases the level of PD-L1 [39]. Kleffel et al. [32] also demonstrated a positive influence of PD-1 on the phosphorylation of ribosomal protein S6 (RPS6), a downstream signaling target of mTOR and marker of its activity, which is associated with poor prognosis in renal cancer [40] and many other cancers [41,42]. In our study, nivolumab did not change the level of pS6 in three cell lines. We detected higher S6 activity levels after nivolumab treatment only in Caki-1 cells. Moreover, contrary to PD-L1 and PD-1 expression, the increase in S6 was noticed at higher concentrations of nivolumab treatment. Additionally, we could not detect PD-L1 protein in the ACHN cell line in the presence of pS6 and increasing levels of PD-1. To implicate this signaling pathway in the treatment of RCC, additional investigations, such as determining the PD-L1 promoter activity or the perturbance of PD-L1 degradation, are planned.

The analysis of AKT activity revealed that in ACHN cells, the ratio of pAKT/AKT was decreased, while in Caki-1 cells, this ratio was elevated. The significance of AKT activity in tumor development is controversial. Since some investigators indicate a relationship between pAKT, poor differentiation and lymph node (LN) metastasis [43], others provide evidence for the association of pAKT with high differentiation and no LN metastasis [44]. In addition, the correlation of pAKT with PD-1 was also investigated. Wang et al. [8] observed an increase in pAKT after the knockdown of PDCD1 (PD-1 coding gene) and a decrease after overexpression by stable phosphorylation of the S6 protein.

In their analysis of diffuse large B-cell lymphoma (DLBCL), Dong et al. [45] described a correlation between PD-L1 and pAKT expression with clinicopathological characteristics. The detection of both proteins in tumors significantly reduced the positive prognosis of the patients compared with patients whose tumors expressed only one of the proteins. The authors suggest using a combination of PD-1/PD-L1 antibodies together with AKT/mTOR inhibitors as a novel therapeutic approach for DLBCL. In our study, three cell lines (Caki-1, RCC30 and RCC31) express both proteins when by ACHN PD-L1 is absent.

In their manuscript, Guan et al. [24] proposed that androgens may be responsible for therapy resistance in advanced prostate cancer patients. This hypothesis is based on the observation that the blockade of both AR and PD-L1 reduces tumor growth and that the inhibition of AR improves T-cell function. Our previous analysis showed differences in the frequency of AR-FL expression and that of its splice variant between ccRCC and normal tissues. We also observed an increased AR expression in pRCC compared with ccRCC [21]. Our current study confirms this observation but only for the in-house established cell lines. In our case, the cell line with the highest AR expression was pRCC30.

In further experiments, we tested the abilities of the cell lines to form aggregates. Both clear-cell carcinoma cells and ACHN cells formed masses with low cell-cell adhesion, but only RCC30 cells formed spheroids whose growth was dependent on nivolumab. In 2D culture, RCC30 displayed higher expression of AR than the other cell lines. However, still no evidence indicates a relationship between AR and spheroid formation. Nevertheless, Niture et al. [46] described an increased ability of prostate cancer cells to create spheroids after microRNA-99b-5p silencing, which was accompanied by the upregulation of the AR axis. However, the relationship between AR expression and spheroid formation remains open. Another aspect is that the growth of cells in a 3D structure may enforce morphological and transcriptional changes or hinder the diffusion of nutrients or drugs (such as nivolumab), which can influence protein expression. These questions require further investigation. Transcriptome analysis of the treated spheroids is the next step to analyze the influence of nivolumab on RCC cells.

As mentioned previously, some patients treated with an immune checkpoint inhibitor (ICI) therapy experience immune-related adverse events (irAEs), which can induce atypical responses, such as hyperprogressive disease (HPD) or pseudoprogressive disease [4,5,6]. A protein implicated in the prevention of the cytotoxic effects of many drugs is cathepsin B [47]. Increased expression of cathepsin B in RCC indicates its role in tumor progression [48]. Our results show that even if Cath B is expressed and active (except in ACHN cells) in RCC, nivolumab did not influence the expression or activity of this protein. This suggests its limited role in processes initiated by this immune checkpoint inhibitor blockade.

According to the S3 guideline renal cell carcinoma (S3-Leitlinie Nierenzellkarzinom) and international guidelines [49] nivolumab can be used as the first-line medicament in combination with other medicaments (ipilimumab, cabozantinib) or as the second-line therapy after the failure of the VEGF/R- or mTOR-based treatment. It is known that VEGFR TKIs affect the protein expression pattern of tumor cells [25] and metabolic cargo and activity in small extracellular vesicles of RCC [50]. Chen et al. [25] describe increased Cath B expression as a response to TKI. In our study with nivolumab, we did not notice any changes in Cath B expression. Our analysis, however, was performed as a simulation of the monotherapy without previous treatment. It is an intriguing question if previous treatment of our cells with TKI would induce similar changes and if further incubation or coincubation with nivoumab would reduce such differences. This question should be addressed in future studies.

## 5. Conclusions

This study clearly shows the intrinsic expression of PD-1 and PD-L1 in clear cell and papillary RCCs and their complex response to nivolumab treatment. Since pRCC cells reacted with elevated tumorigenicity and augmented PD-1/PD-L1 levels and ccRCC reacted with decreased tumorigenicity, further improvements in nivolumab-based therapy for renal carcinoma are needed.

## 6. Limitations

We must address some limitations to our study. Our analysis was performed in vitro, using commercial and in-house established cell lines. For a better validation of our results, the analysis of probes from patients treated with nivolumab with a combination of other medicaments or as the second-line monotherapy should be performed. To implicate signaling pathways in the treatment of RCC, additional investigations, such as determining the PD-L1 promoter activity or the perturbance of PD-L1 degradation, are planned.

## Figures and Tables

**Table 1:** Primers used in qPCR. (*—[28], **—[29]).

Target	Primer	Product Length (bp)
AR-FL *	F: CAGCCTATTGCGAGAGAGCTG	73
	R: GAAAGGATCTTGGGCACTTGC	
AR-V1	F: AGGGAAAAAGGGCCGAGCTA	185
	R: TCCTCCGAGTCTTTAGCAGC	
AR-V3	F: AAGAGCCGCTGAAGGGAAAC	199
	R: AGGCAAGTCAGCCTTTCTTCA	
AR-V4	F: CTCTCAGCTGCTCATCCACA	74
	R: GGTTTTCAAATGCAGCCAGGA	
AR-V7 **	F: AAAAGAGCCGCTGAAGGGAA	150
	R: GCCAACCCGGAATTTTTCTCC	
β-Actin	F: ATTGCCGACAGGATGCAGAA	150
	R: GCTGATCCACATCTGCTGGAA	

## Data Availability

Not applicable.
